# Whole‐Tumor Clearing and Imaging of Intratumor Microbiota in Three Dimensions with miCDaL Strategy

**DOI:** 10.1002/advs.202400694

**Published:** 2024-10-08

**Authors:** Yuezhou Wang, Zile Jiang, Kai Zhang, Huimin Tang, Guimei Wang, Jinshan Gao, Guanghui He, Baoyue Liang, Li Li, Chaoyong Yang, Xianming Deng

**Affiliations:** ^1^ State Key Laboratory of Cellular Stress Biology State‐province Joint Engineering Laboratory of Targeted Drugs from Natural Products School of Life Sciences Faculty of Medicine and Life Sciences Xiamen University Xiamen Fujian 361102 China; ^2^ Department of Infectious Diseases and Hepatology Xiang'an Hospital of Xiamen University School of Medicine Xiamen University Xiamen Fujian 361102 China; ^3^ Cancer Center and Department of Breast and Thyroid Surgery Xiang'an Hospital of Xiamen University School of Medicine Xiamen University Xiamen Fujian 361102 China; ^4^ Department of Pathology Xiang'an Hospital of Xiamen University School of Medicine Xiamen University Xiamen Fujian 361102 China; ^5^ The MOE Key Laboratory of Spectrochemical Analysis and Instrumentation the Key Laboratory of Chemical Biology of Fujian Province State Key Laboratory of Physical Chemistry of Solid Surfaces Department of Chemical Biology College of Chemistry and Chemical Engineering Xiamen University Xiamen Fujian 361005 China; ^6^ Institute of Molecular Medicine Renji Hospital School of Medicine Shanghai Jiao Tong University Shanghai 200127 China; ^7^ Department of Hematology The First Affiliated Hospital of Xiamen University Xiamen University Xiamen Fujian 361003 China

**Keywords:** 3D imaging, centimeter‐scale imaging depth, fluorescent D‐amino acid‐based probes, intratumor microbiota, tissue clearing

## Abstract

Acquiring detailed spatial information about intratumor microbiota in situ is challenging, which leaves 3D distributions of microbiota within entire tumors largely unexplored. Here, a modified iDISCO‐CUBIC tissue clearing and D‐amino acid microbiome labeling‐based (miCDaL) strategy are proposed, that integrates microbiota in situ labeling, tissue clearing, and whole‐mount tissue imaging to enable 3D visualization of indigenous intratumor microbiota. Leveraging whole‐mount spatial resolution and centimeter‐scale imaging depth, the 3D biogeography of microbiota is successfully charted across various tumors at different developmental stages, providing quantitative spatial insights in relation to host tumors. By incorporating an immunostaining protocol, 3D imaging of the immunologic microenvironment is achieved in both murine and human mammary tumors that is previously assumed to be bacteria‐free. Notably, immune infiltrates, including T cells and NK cells, and tertiary lymphoid structures are conspicuously absent in bacteria‐colonized regions. This 3D imaging strategy for mapping Indigenous intratumor microbiota offers valuable insights into host–microbiota interactions.

## Graphical abstract

A modified iDISCO‐CUBIC tissue clearing‐ and D‐amino acid‐based microbiota labeling (miCDaL) strategy is provided, coupled with light sheet fluorescence microscopy, for 3D visualization of intratumor microbiota in relation to the tumor microenvironment, both in a spontaneous murine mammary tumor model and in human breast cancer samples, penetrating centimeter‐thick tumor tissues.

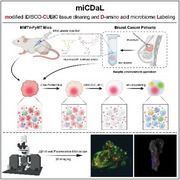

## 1 Introduction

Extensive research on gut microbiota in the past two decades has revealed their diverse functions in human diseases, such as chronic inflammation, autoimmune diseases, cardiovascular disease, and metabolic syndrome.^[^ ^1^ ^]^ Recent studies have uncovered that at least 30 types of cancer coexist with microbiomes.^[^ ^2^ ^]^ This “intratumor microbiota”, characterized by low biomass, is often intracellularly present in both cancer and immune cells within the tumor microenvironment (TME).^[^ ^2^, ^3^ ^]^ Evidence from animal models and clinical studies suggests that intratumor microbiota play significant roles in cancer progression,^[^ ^4^ ^]^ metastasis,^[^ ^5^ ^]^ prognosis,^[^ ^6^ ^]^ and chemoresistance,^[^ ^7^ ^]^ making them increasingly important for diagnostic and prognostic purposes. Consequently, visualizing the whole‐mount, 3D distribution of the intratumor microbiota within the TME is crucial for an unbiased assessment of entire specimens and our understanding of the complex roles these microbes play. However, the primary methods for studying this unique microbiome, including DNA sequencing, imaging of thin‐sectioned tissue slides, and analysis of dissociated single cells, can't fully capture the bacterial quantity, spatial distribution of intratumor microbiota, or specific host‐microbial interactions in an intact tumor.

To visualize the indigenous intratumor microbiota, most of which are not yet amenable for in vitro culture or genetic engineering, in centimeter‐scale tumor tissues, however, poses a great challenge. Histological methods, involving slicing, staining, imaging, and 3D reconstruction of tissue information, are arduous and time‐consuming when mapping extensive tissues.^[^ ^8^ ^]^ These techniques frequently result in mechanical distortion and unintentional loss of tissue sections. Instrument‐based technologies like positron emission tomography or magnetic resonance imaging, commonly used for in vivo multimodal image analysis, fall short in providing the necessary spatial resolution to distinguish specific cellular parameters.^[^ ^9^ ^]^ Intravital two‐photon/multiphoton microscopy has been utilized to directly visualize the gut microbiome and glioma microbiota ex vivo.^[^ ^10^, ^11^ ^]^ Nevertheless, the spatial resolution and imaging depth of this technique are generally confined to ≈500 µm.^[^ ^11^ ^]^ An ideal approach would enable in situ examination of intratumor microbiota while being compatible with feasible tissue processing and comprehensive molecular characterization of distinct cellular features. This is especially critical for visualizing the microbiome in tumors, which exhibit significant heterogeneity in cellular composition and spatial structure.

To address these challenges for 3D intratumor microbiota imaging, here we integrate the use of iDISCO (immunolabeling‐enabled 3D imaging of solvent‐cleared organs) and CUBIC (clear, unobstructed Brain/Body Imaging cocktails and computational analysis) tissue clearing, with a D‐amino acid‐based microbiome in situ labeling protocol. Utilizing the modified iDISCO‐CUBIC tissue clearing‐ and D‐amino acid‐based microbiota labeling‐facilitated (miCDaL) strategy, in conjunction with light sheet fluorescence microscopy (LSFM), we successfully applied to whole‐mount tumor tissue imaging. This approach enabled us to achieve quantitative 3D visualization of intratumor microbiota, both in a spontaneous murine mammary tumor model and in human breast cancer samples, penetrating centimeter‐thick tumor tissues.

## 2 Results

### 2.1 FDAAs Label Intratumor Microbiota In Situ in Both 2D and 3D Contexts

Recently developed fluorescent D‐amino acids (FDAAs) offer a promising approach for the metabolic labeling of bacterial peptidoglycans (PGNs).^[^ ^12^ ^]^ Since mammalian cells exclusively use L‐amino acids for protein synthesis, the supplied FDAAs can be specifically incorporated into the living bacteria^[^ ^12c^ ^]^ within the mammalian host. FDAAs have been reported for in vivo labeling of gut microbiota by Kasper's group and us.^[^ ^10^, ^13^ ^]^ We envisioned that, as an efficient method to fluorescently tag gut microbes by facial oral gavage, FDAAs‐based labeling strategy might hold great potential for imaging indigenous intratumor microbiota in tissue‐cleared tumors, offering appreciable spatial resolution and imaging depth. In our study, we used two water‐soluble FDAAs, TAMRA‐amino‐D‐alanine (TADA) and Cy5‐amino‐D‐alanine (Cy5ADA), which feature either tetramethylrhodamine (TAMRA) or Cyanine 5 (Cy5) on their side chains to test this hypothesis (Figure S1a, Supporting Information). We initially assessed the feasibility of in situ imaging of FDAA‐labeled intratumor microbiota in the mouse mammary tumor virus‐polyoma middle tumor‐antigen (MMTV‐PyMT) transgenic mice, a spontaneous murine mammary tumor mice model that has been reported for harboring substantial populations of tumor‐resident microbiota.^[^ ^5b^ ^]^ Tissue histology slices from the tumor tissue of PyMT mice received an intratumoral injection of TADA 18 h in advance, and showed robust TADA‐positive bacteria signals, which was confirmed by using EUB338, a universal probe against bacterial 16S ribosomal RNA (Figure S1b, Supporting Information, upper). Magnified images revealed the presence of micron‐scale bacteria, indicated by the colocalized signals of both TADA and EUB338 (Figure S1b, Supporting Information, below). Further analyses of dissociated tumor cells from PyMT mice identified these micron‐scale, TADA‐labeled bacteria as clustered punctate dots situated near the perinuclear region of the cells (Figure S1c, Supporting Information). This observation indicates that the indigenous intratumor microbiota can be directly labeled in situ through an intratumoral injection of TADA.

To test whether the FDAAs‐labeled approach is compatible with tissue‐clearing protocols, which render organs transparent to reduce light scattering and increase the imaging depth, we sought for tissue clearing protocols that could preserve the pre‐introduced FDAA signal while ensuring compatibility with antibody immunostaining. We first assessed the hydrophilic CUBIC method^[^ ^14^ ^]^ by administering FDAAs intratumorally in PyMT mice to label native intratumor microbes. Following a 12 h period of treatment, the mice underwent anesthesia and cardiac perfusion with cold phosphate‐buffered saline (PBS) and 4% w/v paraformaldehyde (PFA) in PBS to remove blood. After 48 h of PFA fixation at 4 °C, we observed clear lung tissues post‐CUBIC reagent treatment, though tumor tissues remained opaque (Figure S2a, Supporting Information). This opacity is likely due to the dense fat and structural heterogeneity typical of mammary tumors. We then explored the organic solvent‐based iDISCO protocol using a dichloromethane/methanol gradient for fat removal,^[^ ^15^ ^]^ which significantly enhanced tumor tissue clarity compared to CUBIC (Figure S2b, Supporting Information). Aiming for fully transparent intact tumor tissues suitable for 3D imaging, we applied a CHAPS/NMDEA solution,^[^ ^16^ ^]^ a detergent combination known for deeper tissue penetration than SDS (sodium dodecyl sulfate) and triton X‐100 used in traditional iDISCO protocols, to remove lipids and residue heme from tissues before methanol gradient dehydration. Furthermore, to eliminate the hydrophilic light‐scattering substances, we incorporated a CUBIC‐L reagent clearing step post‐permeabilization (Figure S2c, Supporting Information), the samples were then dehydrated through a methanol gradient to dichloromethane, before transitioning to DBE (Dibenzyl ether) for additional clearing and refractive index matching. This modified iDISCO‐CUBIC approach successfully rendered mammary tumors completely transparent in DBE (Figure S2d, Supporting Information). To demonstrate the specificity of FDAAs for labeling intratumor microbiota in cleared tumor tissues, we employed our miCDaL strategy to perform 3D imaging of mouse PyMT tumors using light sheet fluorescence microscopy (LSFM).^[^ ^17^ ^]^ We observed that antibiotic cocktail (ATBx) administration effectively eradicated intratumor microbiota, as indicated by the significantly reduced fluorescence signals from EUB338 and TADA in comparison to the ATBX(‐)/TADA(+) group (Figure S3a–c, Supporting Information). Notably, in the ATBX(+)/TADA(+) group, the TADA signal was more attenuated than the EUB338 signal (Figure S3c, Supporting Information). This difference is likely due to TADA's ability to label metabolically active bacteria, whereas EUB338 stains both live and dead bacterial DNA following antibiotic treatment. Furthermore, the magnified 3D views of the ATBX(+)/TADA(+) group revealed co‐localized EUB338 and TADA signals, pinpointing the presence of micron‐scale bacterial signals within the cleared tumor tissue (Figure S3c, Supporting Information, enlarged). These findings collectively underscore the specificity of FDAAs probes in situ labeling of intratumor microbiota in 3D contexts.

### 2.2 3D Imaging of Intratumor Microbiota Within Entire Tumors at Different Developmental Stages

**Figure 1:**
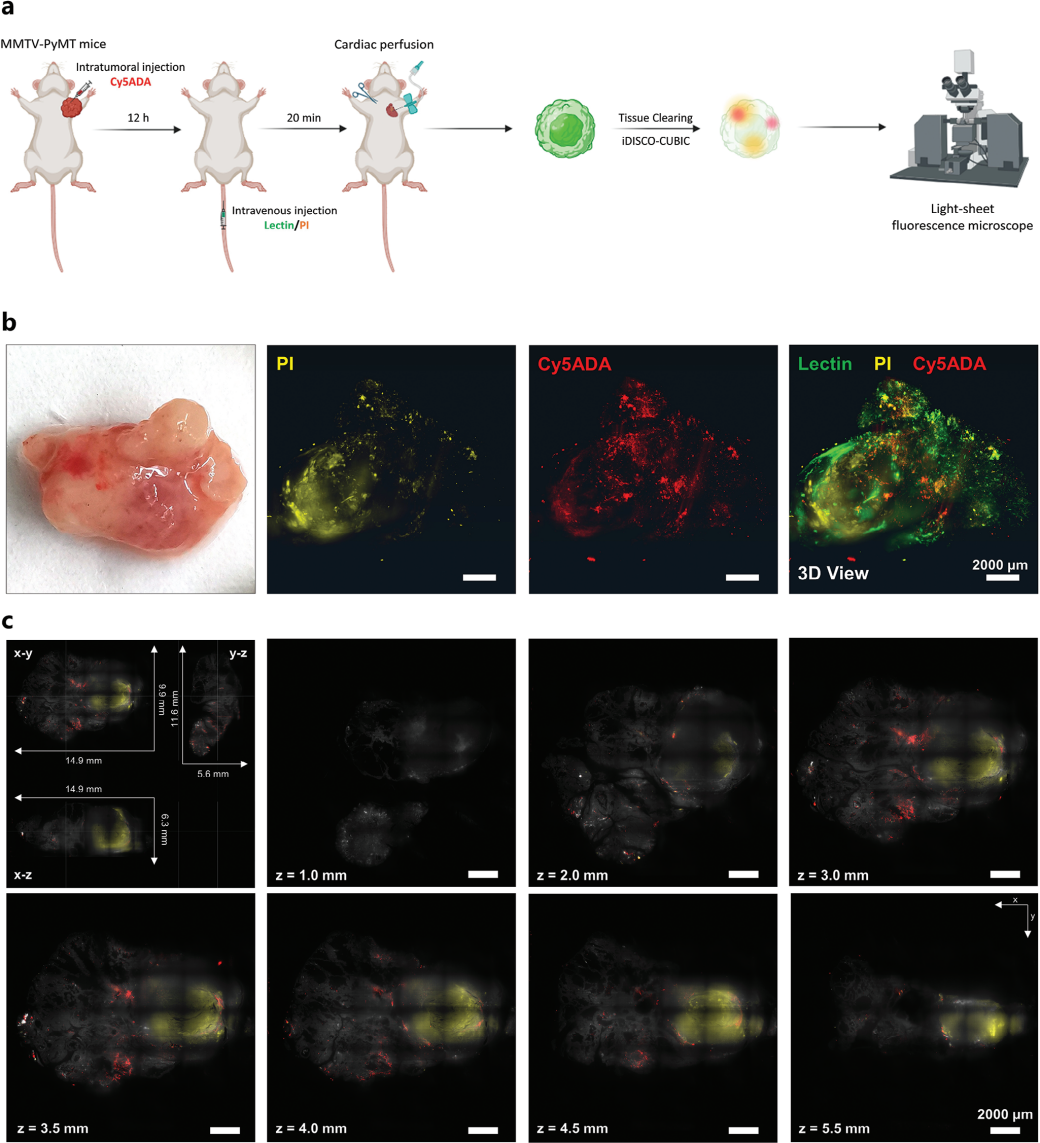
Comprehensive intratumor microbiota mapping in whole‐tumor scale using miCDaL. a) Schematic diagram showing the workflow of 3D imaging of native intratumor microbiota in mammary tumors spontaneously developed in MMTV‐PyMT mice. b) 3D illustration of a whole tumor from MMTV‐PyMT mice. Intratumoral injected Cy5ADA was used for in vivo labeling of intratumor microbes, and intravenous injected propidium iodide (PI) and lectin were used to indicate tumor necrotic area and draw the tumor outline, respectively. Green: Lectin, Yellow: PI, Red: Cy5ADA. Scale bars, 2000 µm. c) The view of the whole tumor from X–Y, X–Z, Y–Z direction, and X‐Y optical sections obtained at 1.0, 2.0, 3.0, 3.5, 4.0, 4.5, and 5.5 mm in Z‐depth. Grey: Lectin, Yellow: PI, Red: Cy5ADA. Scale bars, 2000 µm. In (b) and (c), representative results from three independent experiments are shown.

To delineate the spatial relationship between the intratumor microbiome and specific tumor regions, we administered DL488‐conjugated lectin and propidium iodide (PI) intravenously 20 min prior to mouse anesthesia. This procedure outlined the tumor^[^ ^18^ ^]^ and identified necrotic regions,^[^ ^19^ ^]^ respectively (**Figure** **1**a). The LSFM, in obtaining 3D images of tumors from MMTV‐PyMT mice, distinctly depicts Cy5ADA‐labeled native intratumor microbiota juxtaposed with PI‐stained necrotic regions within the tumor (Figure 1b). Our miCDaL strategy‐facilitated imaging achieved a whole‐tissue spatial resolution and penetrating imaging depth on a centimeter scale (Figure 1b; Video S1, Supporting Information). X–Y optical sections obtained at 1.0, 2.0, 3.0, 3.5, 4.0, 4.5, and 5.5 mm in Z‐depth revealed that the large part of intratumor microbes don't co‐localize with the necrotic core, only a minor fraction of these microbes were found surrounding the necrotic core (Figure 1c). Overall, the 3D reconstructions courtesy of LSFM yield unequivocal comprehensive data on the biogeography of the native microbes within the centimeter‐thick tumor tissues.

**Figure 2:**
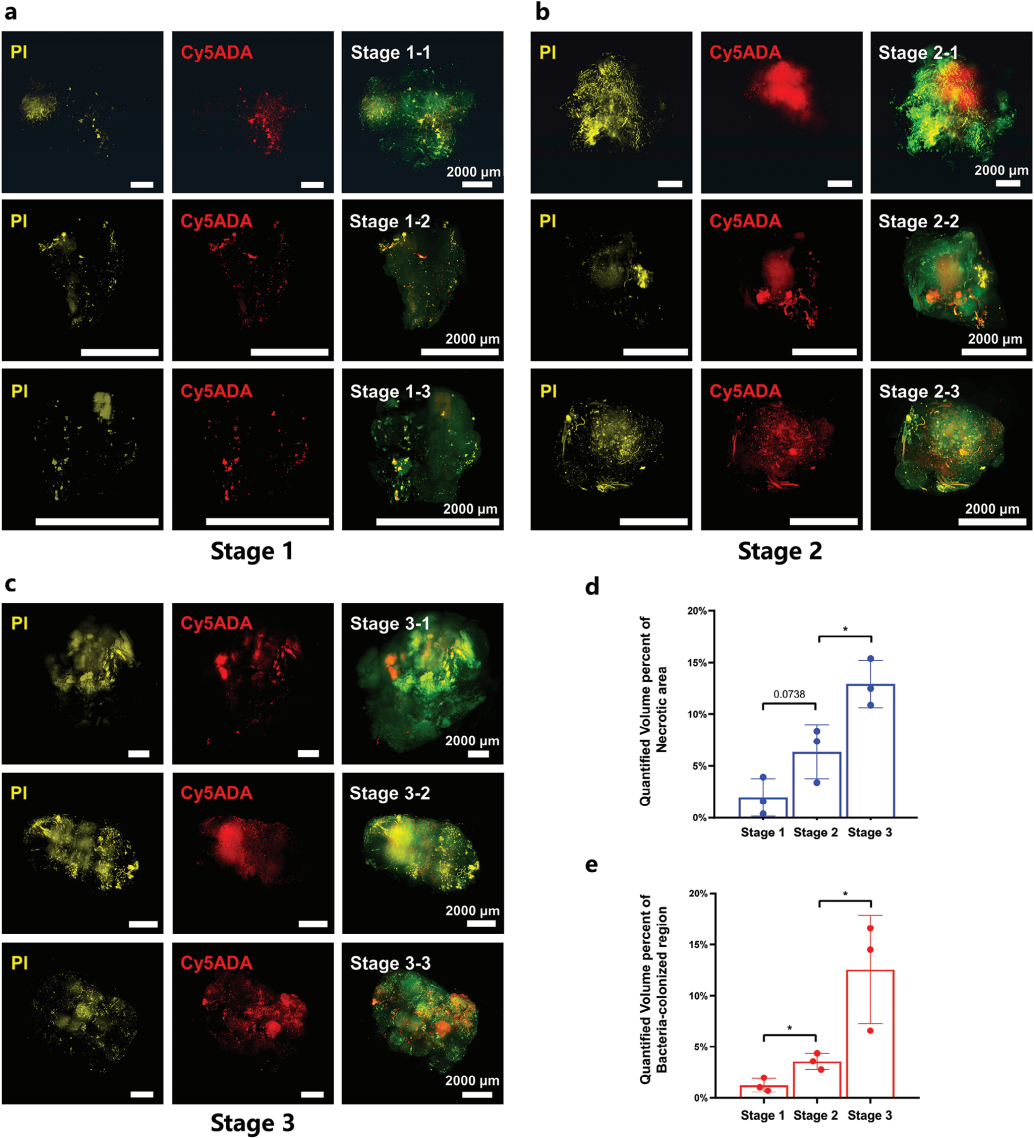
Quantitative analysis of intratumor microbiota in whole tumors at various developmental stages. **a–c)** 3D illustration of tumors at different developmental stages (1‐3) from MMTV‐PyMT mice intratumorally injected with Cy5ADA. *n* = 3 for each stage. Green: Lectin, Yellow: PI, Red: Cy5ADA. Scale bars, 2000 µm. **d)** The quantified volume percentage results of necrotic regions and **e)** bacteria‐colonized regions regarding PyMT tumors in stages 1–3. *n* = 3, data were expressed as mean ± SD. *p* value by a two‐tailed unpaired *t*‐tests. ^*^ *p *< 0.05.

To explore the usefulness of our developed miCDaL protocol, we investigated the quantity and spatial distribution of bacteria within intact tumors at various stages of tumor development. Using the imaging protocol described above (Figure 1a), we sampled and imaged three tumors at each developmental stage (1–3). The tumors in stage 1 displayed a pale appearance (Figure S4a, Supporting Information) with no significant signs of vascular genesis and scattered necrotic areas in the 3D views (**Figure** **2**a; Videos S2–S4, Supporting Information), indicating of an early developmental stage. In contrast, tumors at stages 2 and 3 exhibited marked angiogenesis (Figure S4a,b, Supporting Information) and layered necrotic regions (Figure 2b,c), suggesting more advanced stages. Particularly, stage 3 tumors showed more extensive necrosis than those in stage 2, as volumetrically quantified necrotic regions demonstrated (Figure 2d). Additionally, in the 3D views of stage 2 and stage 3 tumors, the vascular morphology and microvessel diameters varied significantly, ranging from 50 to 450 µm (Figure S4b,c, Supporting Information). This indicates high heterogeneity in the 3D vascular patterns across different tumor stages. Subsequently, we volumetrically quantified the bacteria‐colonized regions. This involved counting the total number of Cy5 voxels within the tumor and multiplying by the volume of a single Cy5 voxel. Our 3D quantitative analysis revealed that the proportion of indigenous microbial communities within the tumor increases as the tumor progresses, evidenced by the expansion of necrotic regions (Figure 2d) and a corresponding rise in microbial presence (Figure 2e).

We then examined the spatial relationship between intratumor microbiota and necrotic regions across the entire tumor scale. For tumors in stage 1, the 3D imaging results showcased in Figure 2a and the X–Y optical sections at Z‐depths (Figure S5a, Supporting Information) revealed a pattern similar to that observed in Figure 1b. At this stage, necrotic areas were predominantly dispersed, with only a minor fraction of the microbiome colocalizing with these necrotic zones (Videos S2–S4, Supporting Information). In contrast, for tumors in stages 2 and 3, (Figure 2b,c; Videos S5–S10, Supporting Information), the PI‐stained necrotic cores—located centrally within the tumors—completely enveloped the regions colonized by bacteria. This finding is further supported by X–Y optical sectioning at multiple Z‐depths (Figure S5b,c, Supporting Information). These observations suggest that the intratumor microbes exhibit a preference for necrotic regions, which are often hypoxic,^[^ ^20^ ^]^ in advanced tumor stages.

### 2.3 3D Imaging Demonstrates the Presence of Microbiota in Lung Metastasis Foci

In the advanced stages of PyMT tumor growth, most mice with tumors also develop lung metastases. A recent study has reported that cancer cells can metastasize alongside their intracellular bacteria, the bacteria‐positive circulating tumor cells can colonize and shape the metastasis in the lung.^[^ ^5b^ ^]^ We explored whether FDAAs could probe the microbiome within these metastatic lung tissues in vivo, focusing first on its feasibility in thin tissue sections, which revealed multiple metastatic foci (Figure S6a, Supporting Information). To label the indigenous microbiome in the lung, we used an intravenous injection of TADA. At 6 h post‐injection, the harvested lung tissues were subjected to fixation and methanol processing to eliminate any unbound TADA. We found that TADA signals and the 16S FISH EUB338 probe were co‐localized in the whole‐mount lung tissue section, and most of the identified bacteria were enriched in the metastatic foci (Figure S6b, Supporting Information), thus validating the effectiveness of intravenous TADA injection for labeling the indigenous lung microbiome.

**Figure 3:**
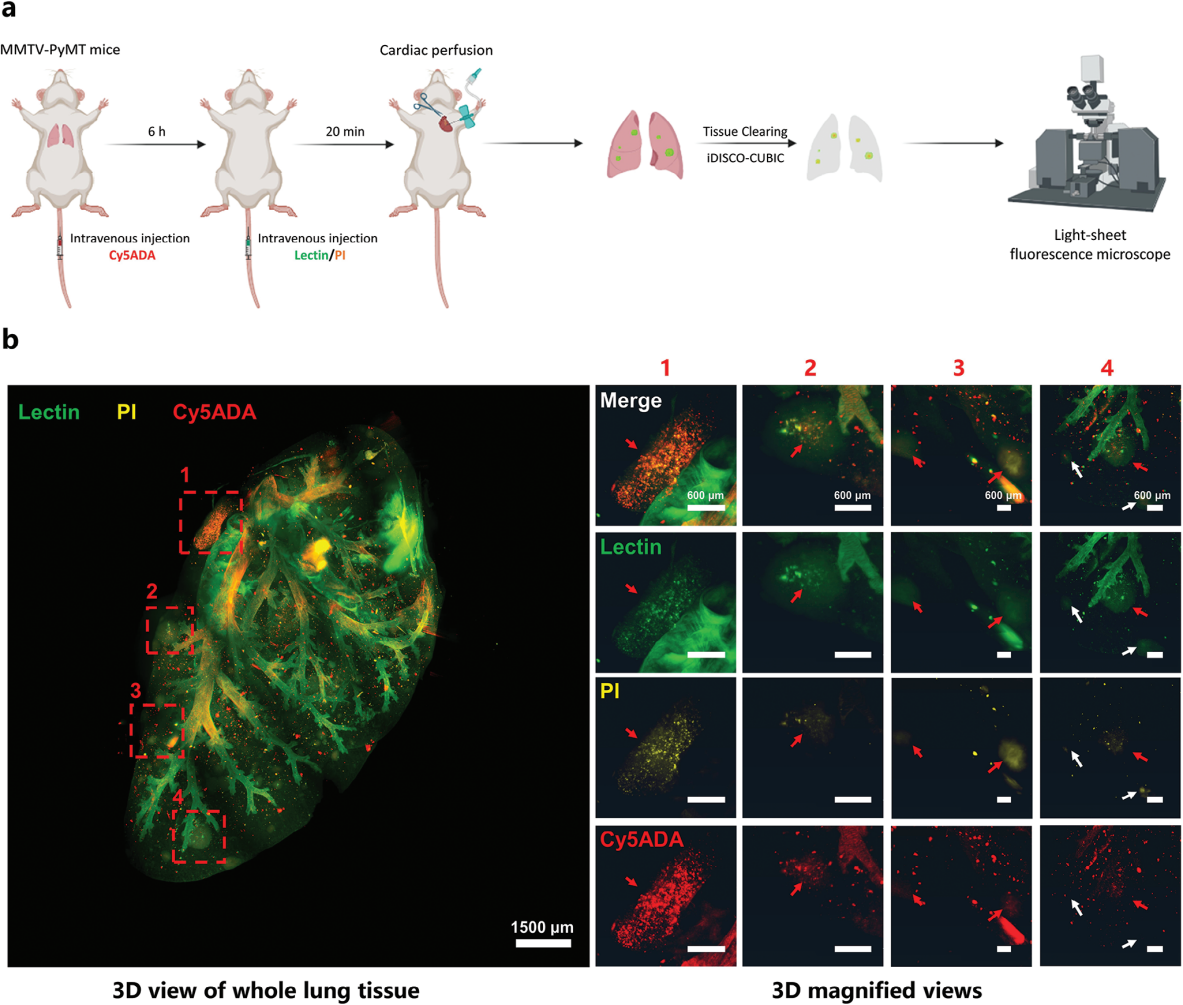
3D imaging of indigenous microbiome in whole‐lung tissue using miCDaL. a) Schematic diagram showing the workflow of 3D imaging of indigenous microbiome in lung tissues in MMTV‐PyMT mice. b) 3D imaging of whole lung tissue from MMTV‐PyMT mice. Cy5ADA was used for the in vivo labeling of indigenous microbes. Propidium iodide (PI) and lectin were used to indicate metastatic foci and draw the pulmonary trachea, respectively. Left, the complete field of view of the whole lung. Right, higher magnification views of the regions are marked by the numbered red dashed box. Red arrows indicate colocalized metastatic foci and microbes, and white arrows indicate weak or dispersed bacteria signals within the indicated metastatic foci. Green: Lectin, Yellow: PI, Red: Cy5ADA. Scale bars for whole lung and magnified views are 1500 and 600 µm, respectively. Representative data from three independent experiments are shown.

To get 3D imaging for the lung, we intravenously injected DL488‐conjugated lectin and PI into a PyMT mouse 20 min prior to anesthesia (**Figure** **3**a). The lectin provided a clear depiction of the pulmonary trachea, while the concurrent use of lectin and PI outlined lung metastasis foci of varied sizes within the lung (Figure 3b; Video S11, Supporting Information). Out of seven identified macro‐metastasis foci, with diameters ranging from 600 to 1500 µm, five displayed noticeable bacterial signals as indicated by Cy5ADA (Figure 3b, enlarged). As for the other smaller‐scale metastasis foci, the bacteria signal was weak and dispersed, possibly owing to that a single dose of intravenous Cy5ADA is insufficient to label the complete indigenous microbiome within the entire lung. An alternative explanation could be that not all lung metastasis foci contain bacteria or metabolically active bacteria, which was also observed in the thin section imaging (Figure S6b, Supporting Information). Taken together, we have unequivocally mapped the biogeography of indigenous microbes within lung metastasis foci, offering fresh insights into the microbial interactions within the metastatic environment.

### 2.4 Segregation Between the Intratumor Microbiome and Activated *T*‐Cells in Mouse PyMT Tumors

**Figure 4:**
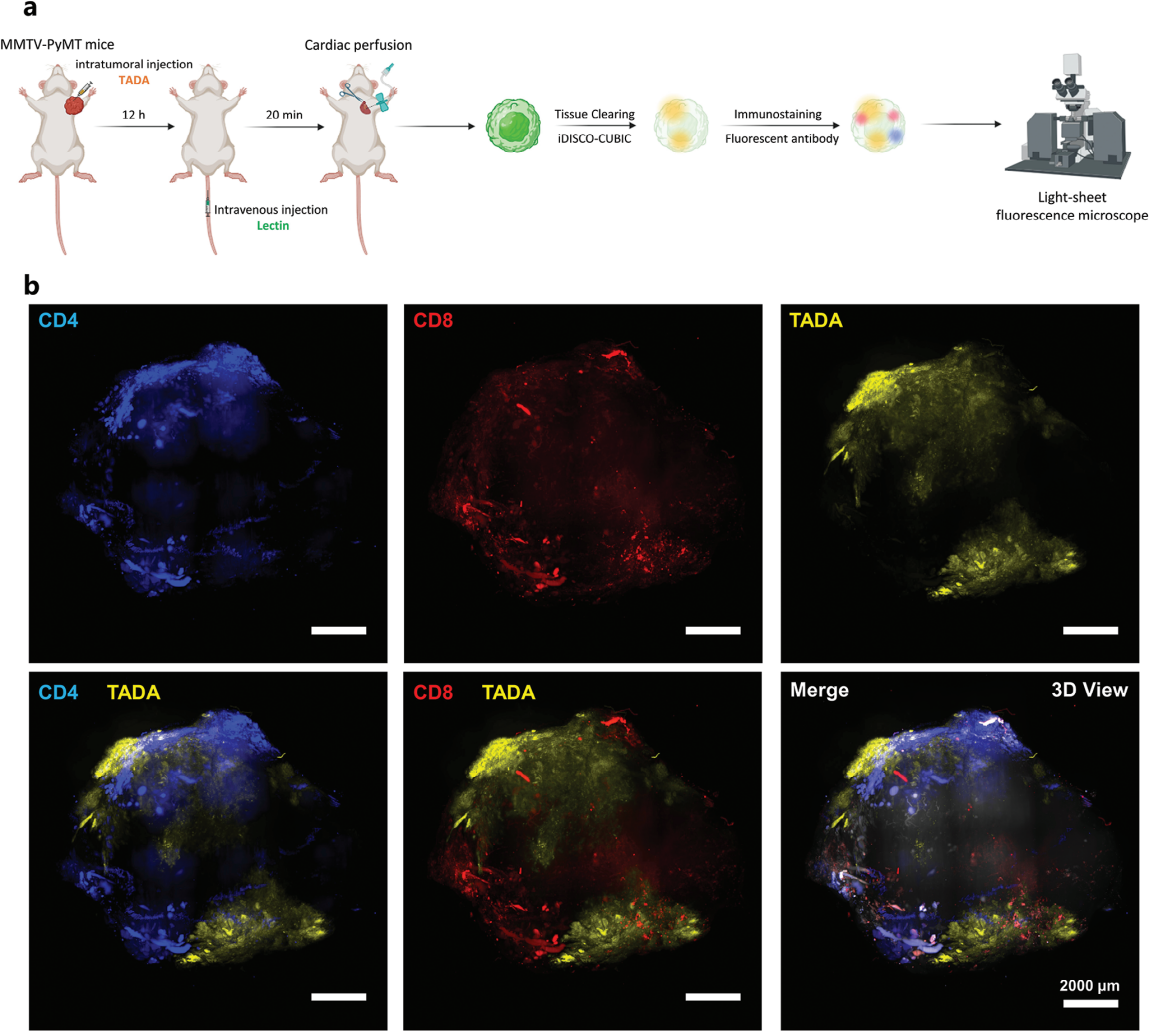
3D views of spatial distribution between intratumor microbiota and activated *T*‐cells. a) Schematic diagram showing the workflow of 3D imaging of native intratumor microbiota and related tumor immune microenvironment by the integrated antibody immunostaining. b) 3D view of a whole tumor from MMTV‐PyMT mice. Activated *T*‐cells are indicated by CD4 and CD8 antibodies. Intratumorally injected TADA was used for the in vivo labeling of intratumor microbes. Blue: CD4, Yellow: TADA, Red: CD8, White: DAPI. Scale bar, 2000 µm. Representative results from three independent experiments are shown.

To further extend the use of our miCDaL strategy, we wondered whether our strategy is compatible with immunostaining by antibodies. Previous studies have shown that in human tissue sections of oral squamous cell carcinoma and colorectal cancer, intratumor microbes tend to prefer the immune suppressive region within the tumor microenvironment.^[^ ^2c^ ^]^ However, the specific bacterial distribution throughout entire tumors remains unclear. Notably, while the oral cavity and colon are recognized for their extensive bacterial colonization, the interaction between immunocyte and intratumor microbiota in traditionally considered bacteria‐free tissues such as breast cancer tissues is still poorly understood. In this experimental set, we intratumorally injected TADA into PyMT mice to label the native intratumor microbes. Followed by the tissue‐clearing process, anti‐CD4 and anti‐CD8 antibodies were introduced during the final stage of permeabilization. After dehydration, 3D imaging was conducted in DBE (**Figure** **4**a). The LSFM‐acquired 3D images clearly showed that TADA‐labeled intratumor microbes predominantly accumulated in two distinct regions within the mammary tumor tissues (Figure 4b; Video S12, Supporting Information). X–Y optical sections, taken at various Z‐depths (1.0, 2.0, 2.5, 3.0, 4.5, and 5.5 mm), revealed a notable concentration of intratumor microbes near the tumor border. In contrast, CD4^+^ T cells were primarily located at the periphery of tumors, exhibiting moderate immune infiltration at Z‐depths of 2.5–5.5 mm. Meanwhile, CD8^+^ T cells demonstrated significant deep tissue penetration in Z‐stacks ranging from 2.5 to 4.5 mm (Figure S7, Supporting Information). Both the 3D and X–Y optical sections results indicate segregation between the intratumor microbiome and the CD4^+^ and/or CD8^+^ compartments, suggesting activated *T*‐cells are excluded in bacteria‐colonized regions within the entire tumor tissue.

### 2.5 TLS and NK Cells are Excluded from Regions Colonized by Intratumor Microbiota in Patient Breast Cancer Tissues

**Figure 5:**
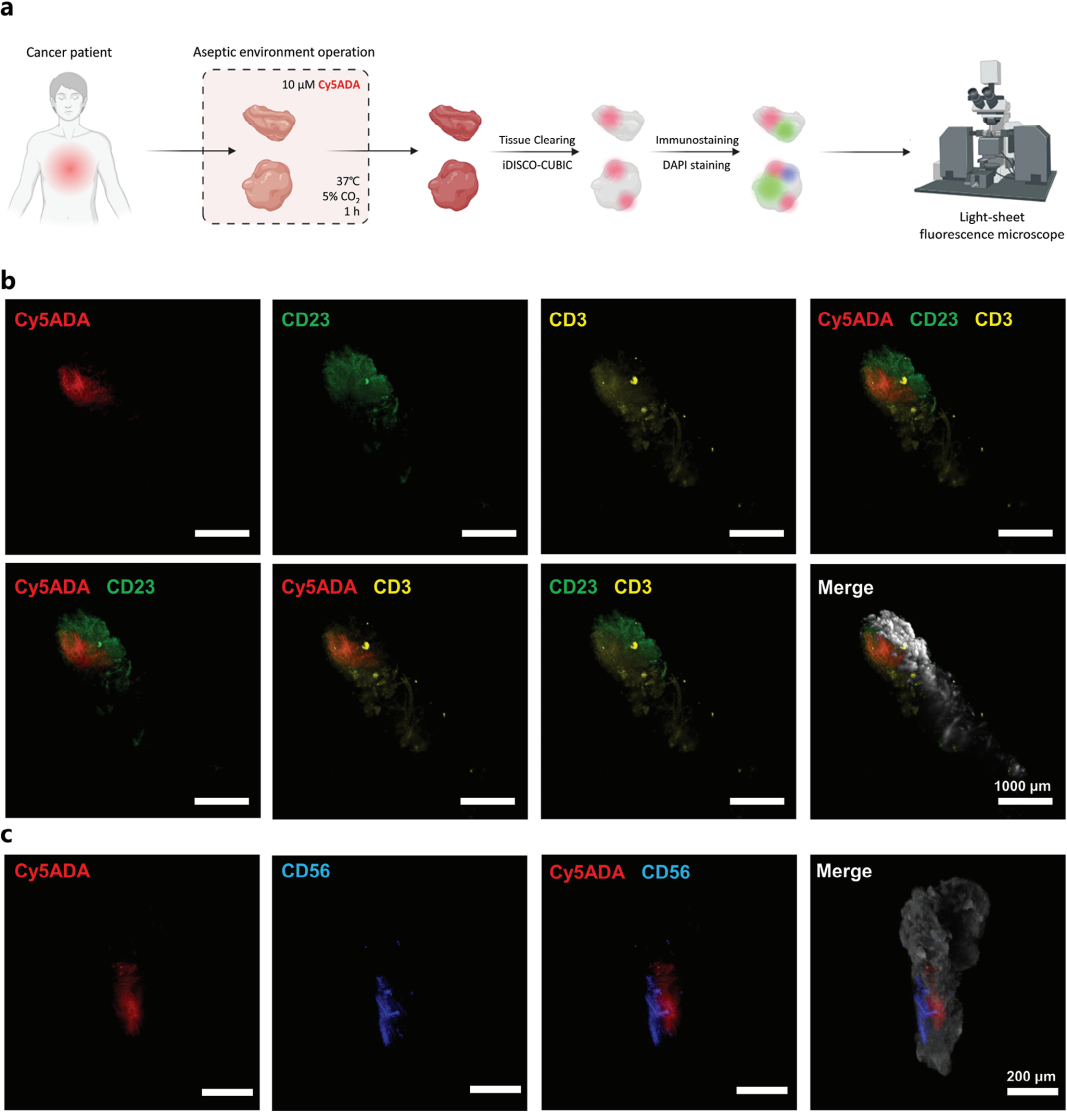
3D mapping of intratumor microbiota and immunocyte infiltration in cancer patient tissues. a) Schematic diagram showing the workflow of 3D imaging of native intratumor microbiota and immunocyte infiltration in breast cancer patient tissues. b) 3D imaging of intratumor microbiota and tertiary lymphoid structures (TLS), c) intratumor microbiota and NK cells in human breast tumor tissues. TLS is indicated by CD3 and CD23 antibodies, and NK cells are indicated by CD56 antibody. Cy5ADA was used for the in vivo labeling of intratumor microbes. Green: CD23, Yellow: CD3, Blue: CD56, Red: Cy5ADA, White: DAPI. Scale bars, 1000 µm in (b) and 200 µm in (c). In (b) and (c), representative results from two independent experiments are shown.

To evaluate the applicability of our method for 3D imaging of human cancer tissues, fresh human breast tumor (BT) tissues, collected under aseptic conditions, were incubated in a serum‐containing dulbeccos modified eagle medium (DMEM) supplemented with Cy5ADA, aimed at labeling the native intratumor microbiota (**Figure** **5**a). Followed by a incubation, patient BT tissues were fixed with methanol to eliminate any residual Cy5ADA. Tissue section analysis of breast cancer patient disclosed a significant presence of the indigenous intratumor microbiome, evidenced by the overlapping signals of Cy5ADA and EUB338 (Figure S8a, Supporting Information).

Tertiary lymphoid structures (TLS), typically absent under normalphysiological conditions, are organized clusters of immune cells that emerge in cancer.^[^ ^21^ ^]^ Notably, TLS can facilitate the infiltration of immune cells such as natural killer (NK) cells into the tumor. Nevertheless, the distribution patterns of TLS and NK cells throughout the entire tumor, in relation to the native intratumor microbiome, remained elusive. Therefore, we next explored the spatial distribution/interaction between TLS and the intratumor microbiome, as well as between NK cells and the intratumor microbiome.

To visualize TLS and NK cells in human BT tissues, we applied our miCDaL strategy, which involves tissue clearing followed by staining. For TLS, we used CD23 antibody (marker for conventional B cells and follicular dendritic cells) and CD3 antibody (*T*‐cell marker). NK cells were stained using CD56 antibodies. Post‐tissue clearing, these two tissues of human breast cancer became sufficiently transparent (Figure S8b, Supporting Information). The 3D imaging results clearly revealed that conventional B cells, follicular dendritic cells, and *T*‐cells form clusters representing TLS within the human BT tissues. Notably, there was minimal overlap between the intratumor microbiota and TLS (Figure 5b; Video S13, Supporting Information). The spatial relationship between NK cells and the intratumor microbiota revealed a segregation pattern (Figure 5c; Video S14, Supporting Information). These results suggest significant immune infiltration within these human BT tissues, yet TLS and NK cells are predominantly excluded from regions colonized by bacteria.

## 3 Conclusion

Visualization of the whole‐mount distribution of the intratumor microbiota is a challenge for the study of roles of microbiomes in tumor development. Here we developed a miCDaL strategy for 3D imaging of indigenous intratumor microbiota through selectively labeling living bacteria by intratumorally administered FDAAs. The visualization of these unique microbes in the centimeter‐scaled tumor tissues, provides the whole‐mount spatial information of these highly complex microbes in a 3D manner. Furthermore, the quantitative analysis capability of our strategy offers an opportunity for understanding of the dynamic interaction between intratumor microbiota and the evolving necrotic area during tumor development. An increasing proportion of indigenous intratumor microbiota, along with expanded necrotic areas, might serve as diagnostic markers for advanced‐stage tumors. The potential tumor‐promoting effects of intratumor microbiota^[^ ^4^, ^5^, ^22^ ^]^ highlight them as promising therapeutic targets; accordingly, antibiotics that target these microbiota could offer protective benefits to cancer patients.^[^ ^23^ ^]^ Our miCDaL strategy is also compatible with immunostaining protocols, enhancing our understanding of the spatial distribution of intratumor microbiota and its relationship with the surrounding tumor microenvironment. To achieve better imaging results, an improved tissue clearing and immunostaining protocol that can preserve the intricate microstructure of entire tumors and improve antibody penetration is required, particularly for the human cancer tissues,^[^ ^24^ ^]^ in which the necrotic core is challenging for 3D in‐depth imaging. Moreover, to investigate the interactions between different bacterial genera, taxonomically recognizing bacteria in whole tumors with techniques that are potentially compatible with our miCDaL strategy, such as FISH staining, may be further examined. Overall, our miCDaL strategy introduces a promising strategy for 3D imaging of indigenous intratumor microbiota. We anticipate that this capability to visualize intratumor microbes in their native context, with whole tumor spatial resolution and centimeter imaging depth, will be pivotal in gaining new insights into host‐microbe interactions within the natural tumor microenvironment.

## 4 Experimental Section

### Reagents

Two fluorescent D‐amino acids (FDAAs) probes, TAMRA‐amino‐D‐alanine (TADA) and Cy5‐amino‐D‐alanine (Cy5ADA), were purchased from Shanghai Biochempartner Co., Ltd. (Shanghai, China). FISH probes and paraformaldehyde were from Sangon Biotech (Shanghai, China). Other chemicals, not noted above, were from Sigma‐Aldrich (St. Louis, MO, USA).

### Mice

Female MMTV‐PyMT transgenic mice (FVB/N‐Tg(MMTV‐PyVT)634Mul/J, 002374), which develop spontaneous breast tumor, were kind gifts from Prof. Sheng‐Cai Lin's lab (School of life sciences, Xiamen university), originally from The Jackson Laboratory (Cat#002374), and bred in the Laboratory Animal Center of Xiamen University. During the entire study period, mice were housed on a standard condition, with a temperature of 23–26 °C, a controlled 12 h/12 h light/dark cycle, and humidity of 55%, with free access to food and water. All animal care and experimental procedures complied with the guidelines from the Institutional Animal Care and Use Committee at the Experimental Animal Centre at Xiamen University.

### Mice Sample Collection

Tumor‐bearing mice were housed in the SPF‐level Laboratory Animal Center at Xiamen University. The cage, feed, pad, etc., were sterilized by high‐pressure steam and replaced regularly. The water‐soluble TADA and Cy5ADA were sterilized using a 0.22 micron filter membrane. After the intratumoral injection of TADA/Cy5ADA, tumor dissection, and processing were meticulously performed in a clean, sterile cell culture hood using autoclaved dissection tools.

### Antibiotics Treatments on MMTV‐PyMT Mice

To specifically eliminate intratumor microbiota, MMTV‐PyMT mice were administered an antibiotic cocktail (ATBx) as described^[^ ^5b^ ^]^ with some modifications, mice were administered with high dose ATBx (300 µL/mouse), containing vancomycin (50 mg mL^−1^; Sangon Biotech), imipenem/cilastatin (25 mg mL^−1^; Merck Sharp&Dohme Corp.U.S.A), neomycin (10 mg mL^−1^; Sangon Biotech)) by intravenous injection daily for five to seven consecutive days.

### Confocal Fluorescence Microscopy

Confocal fluorescence imaging was performed on an orthotopic laser confocal microscopy Zeiss LSM 900+Airyscan2 (Zeiss, Germanry). Whole tumor/lung sections were imaged with 10x objective lens (0.45 NA) in tile mode (Imaging setup: Frame Size 1024×1024, Zoom 1×, Pixel time 0.52 µs, Averaging 1, 16 bits per Pixel, 1 AU (Airy Units), bilateral scanning). Cells, yeast, bacteria, and local tumor sections were imaged with 63x oil immersion lens respectively (1.4 NA) (Imaging setup (cells, yeast, local tumor sections): Frame Size 1437×1437, Zoom 1×, Pixel time 0.54 µs, Averaging 2, 16 bits per Pixel, 1 AU (Airy Units), bilateral scanning. Imaging setup (bacteria): Frame Size 287×287, Zoom 5×, Pixel time 0.87 µs, Averaging 4, 16 bits per Pixel, 1 AU (Airy Units), bilateral scanning). All images were background subtracted and contrast was uniformly enhanced.

### 3D Imaging with Light‐Sheet Fluorescence Microscope (LSFM)

After tissue clearing by our modified iDISCO‐CUIBC protocol, 3D imaging was performed on a light‐sheet fluorescence microscope Nuohai LS‐18 (Nuohai Life Science, Co., Ltd., Shanghai, China). Depending on the sample size, choose a 4× (4 tile, imaging resolution: *x* = 3.3 µm, *y* = 3.3 µm, *z* = 7 µm) or 6.3× (6 tile, imaging resolution: *x* = 2 µm, *y* = 2 µm, *z* = 5 µm) objective for image acquisition. Subsequently, the raw imaging data is processed for image reconstruction and the generated image is processed and optimized using Imaris (v. 9.0, Bitplane, Oxford, UK) and Amira image processing software. All images were background subtracted and contrast was uniformly enhanced.

### Labeling of Tumor‐Associated Microbiota with FDAA Probes

FDAAs probe (200 µm, 100 µL) was intratumorally injected into PyMT mice. The mice underwent cardiac perfusion to remove blood and were pre‐fixed 18 h later. The tumor was harvested and washed with PBS, then fixed in 4% PFA at 4 °C.

### Labeling of Lung‐Associated Microbiota with FDAA Probes

FDAAs probe (200 µm, 100 µL) was intravenously injected into PyMT mice. The mice underwent cardiac perfusion to remove blood and were pre‐fixed 6 h later. The lung was harvested and washed with PBS, then fixed in 4% PFA at 4 °C.

### Labeling of Blood Vessels and Tumor Necrotic Areas with Lectin and PI Respectively

DL488 conjugated Lectin (1: 50, 100 µL) and Propidium Iodide (0.2 mg mL^−1^, 100 µL) were intravenously injected into the mice which had been labeled at least 5 min before cardiac perfusion to enable adequate circulation and binding, as previously described.^[^ ^25^ ^]^

### Tissue Clearing Through Modified CUBIC

Tissue clearing was conducted by the CUBIC protocol as described previously^[^ ^14b^ ^]^ with some modifications. Briefly, the tissues were extracted from mice that had undergone cardiac perfusion and initially fixed in 4% PFA at 4 °C for 24 h, followed by subsequent washing with PBS for at least 2 h at room temperature three times. Then the samples were delipidized in a H_2_O/CUBIC‐L (TCI #3740) (1:1) solution for a duration of 6–24 h, followed by a solvent exchange to CUBIC‐L (N‐Butyldiethanolamine: Triton X‐100: Water = 1: 1: 8). Refresh CUBIC‐L on days 1, 2, and every other subsequent day. After delipidation, tissues were washed with PBS for 2 h at room temperature three times, followed by a solvent exchange to H_2_O/CUBIC‐R+ (TCI #3741) (1:1) solution for a duration of 6–24 h. Finally, CUBIC‐R+ (Antipyrine: N‐Methylnicotinamide: N‐Butyldiethanolamine: Water = 90: 60: 1: 29) was used for RI matching.

### Tissue Clearing Through Modified iDISCO

Tissue clearing was conducted by iDISCO+ protocol as described previously^[^ ^15d^ ^]^ with some modifications. Briefly, the mice which was labeled were deeply anesthetized with pentobarbital (≈150 mg kg^−1^ of body weight) and fixed with intracardiac perfusion of ice‐cold 0.9% saline (with 10 mg mL^−1^ heparin) and 4% PFA. All harvested samples were post‐fixed overnight at 4 °C in 4% PFA and then washed with 1 × PBS for 1 h at room temperature twice.

The samples were then dehydrated at room temperature in 20%, 40%, 60%, 80% and 100% methanol for 1 h. After that, they were incubated in 100% methanol for 1 h again, followed by a solvent exchange to bleach using a mixture of 5% H_2_O_2_ in 20% DMSO/methanol (1 vol 30% H_2_O_2_, 1 vol DMSO, and 4 vol methanol) at 4 °C. After bleaching, the samples were re‐equilibrated at room temperature slowly and re‐hydrated in 100%, 80%, 60%, 40%, and 20% methanol for 1 h, and finally in 0.2% for 1 h twice. Pre‐treated samples were then incubated in permeabilization solution (0.2% Triton X‐100, 20% DMSO, 0.3 m glycine, in 0.1 m PBS (pH = 7.4)) at 37 °C for 36 h. Subsequently, the samples were dehydrated in 20%, 40%, 60%, 80%, and 100% methanol for 1 h each, and then incubated in DCM/MeOH (2: 1) until they sank at the bottom of the vial and in 100% DCM for 20 min twice to wash out the methanol. Finally, samples were incubated (without shaking) in DiBenzyl Ether (DBE, Sigma 108014) until clear. The samples were stored in DBE at room temperature.

### Tissue Clearing Through our Modified iDISCO‐CUBIC Protocol—Acquisition

The mice that were labeled were deeply anesthetized with pentobarbital (≈150 mg kg^−1^ of body weight). The mice underwent cardiac perfusion to remove blood and were pre‐fixed. After cardiac perfusion, tissues were harvested and washed with PBS, then fixed in 4% PFA at 4 °C for 24 h.

### Tissue Clearing Through our Modified iDISCO‐CUBIC Protocol—Delipidation

After fixation by 4% PFA, tissues were washed with PBS for 1 h at room temperature twice. The samples were then degreased with 5% CHAPS (CAS.75621‐03‐3) +12.5%(v/v) N‐methyldiethanolamine for 24 h at room temperature.^[^ ^16^, ^21^ ^]^ At the same time, partially coagulated blood was removed which allows partial decolorization of the tissue to reduce autofluorescence

### Tissue Clearing Through our Modified iDISCO‐CUBIC Protocol—Dehydration

The samples were then dehydrated at room temperature in 20%, 40%, 60%, and 80% methanol for 2 h and then incubated in 100% methanol for 2 h twice. The remaining lipids from the previous step can be further removed in this step.

### Tissue Clearing Through our Modified iDISCO‐CUBIC Protocol—Decolorization

To minimize tissue autofluorescence as effectively as possible, the solvent was changed to 5% H_2_O_2_ in 20% DMSO/methanol (1 vol 30% H_2_O_2_/, 1 vol DMSO, 4 vol methanol, ice cold) and the samples were then bleached at 4 °C for 24 h.

### Tissue Clearing Through our Modified iDISCO‐CUBIC Protocol—Permeabilization

After bleaching, the samples were rehydrated in 100%, 80%, 60%, 40%, and 20% methanol and then in PBS for 1 h each and then in permeabilization solution (0.2% Triton X‐100, 0.1% Deoxycholic acid, 100 mm Glycine, 100 mM EDTA, 10% DMSO, 1% heptakis(2,6‐di‐O‐methyl)‐β‐cyclodextrin, in 0.1 m PBS(pH = 7.4),) at room temperature overnight.^[^ ^21a^ ^]^

### Tissue Clearing Through our Modified iDISCO‐CUBIC Protocol—Immunofluorescence Staining (Optional Step)

The treated samples were incubated in blocking solution (0.2% Triton X‐100, 10% DMSO, 5% Donkey serum, in 0.1 m PBS (pH = 7.4)) at 37 °C for 24–48 h. The samples were then immunolabeled with fluorophore‐conjugated primary antibody in labeling solution (0.2% Tween‐20, 10 mg mL^−1^ heparin, 10% DMSO, 5% Donkey serum, appropriate concentration of fluorophore‐conjugated antibody (listed in below, **Table** **1**), in 0.1 m PBS (pH = 7.4)] at 37 °C for at least 72 h. After antibody labeling, the samples were washed four times with wash solution [0.2% Tween‐20, 10 mg mL^−1^ heparin, in 0.1 m PBS (pH = 7.4)], each lasting for a minimum duration of 4 h.

**Table 1:** Antibodies used in direct immunofluorescence assays.

Antibody	Final Concentration	Ex/Em (nm)	Brand	Cat No.
DyLight 488 Labeled Lycopersicon Esculentum (Tomato) Lectin (LEL, TL)	1:1000	488/510	VECTOR	DL‐1174‐1
Brilliant Violet 510 anti‐mouse CD4	1:500	405/510	Biolegend	100449
Alexa Fluor 647 anti‐mouse CD8a	1:500	633/660	Biolegend	100724
PE anti‐human CD56 (NCAM)	1:200	550/570	Biolegend	304605
PE anti‐human CD3	1:200	550/570	Biolegend	300307
FITC anti‐human CD23	1:200	488/520	Biolegend	338506

### Tissue Clearing Through our Modified iDISCO‐CUBIC Protocol—Fluorescence In Situ Hybridization of Bacteria (Optional Step)

The treated samples were washed with 2*SSC (3 m NaCl, 0.3 m Trisodium citrate) for 2 h twice and then incubated in lysis solution (1 mg mL^−1^ lysozyme in 2*SSC (pH = 7.4)) at 37 °C for 12–24 h. The samples were then dehydrated at room temperature in 50%, 70%, 90%, and 98% ethanol for 2 h and then incubated in 100% ethanol for 2 h twice. Before hybridization, the treated samples were incubated in a hybridization buffer (**Table** **2**) without a probe at 46 °C for 24 h, followed by a solvent exchange to hybridization buffer with a probe (5 µg mL). After at least 48 h of hybridization, the samples were washed four times with termination solution (**Table** **3**) at 48 °C, each lasting for a minimum duration of 4 h.

**Table 2:** Hybridization solution preparation scheme.

Stock reagent	Volume	Final concentration
5 m NaCl	360 µL	900 mm
1 M Tris‐HCl (pH = 7.4)	20 µL	20 mm
Formamide	% dependent on probe	
ddH_2_O	Add to 2 mL	
10% SDS	2 µL	0.01%
(add SDS last to avoid precipitation)		

**Table 3:** Termination solution preparation scheme.

%formamide in Hybridization solution	µL 5m NaCl in 50 mL	Concentration of NaCl
0	9000	0.900
5	6300	0.636
10	4500	0.450
15	3180	0.318
20	2150	0.225
25	1490	0.159
30	1020	0.112
35	700	0.080
40	460	0.056
45	300	0.040
50	180	0.028
55	100	0.020
60	40	0.014

### Tissue Clearing Through our Modified iDISCO‐CUBIC Protocol—Treated with CUBIC

After permeabilization, the samples were treated with H_2_O/CUBIC‐L (TCI #3740) (1:1) solution for a duration of 6 h, followed by a solvent exchange to CUBIC‐L. Refresh CUBIC‐L 12 h later and after another 24 h, the samples were washed with PBS for 2 h twice.

### Tissue Clearing Through our Modified iDISCO‐CUBIC Protocol—Agarose Embedding

The sample was further washed with 20% methanol for 2 h. Subsequently, a 2% low melting point agarose solution was prepared and the tissues were embedded once the temperature had cooled down to 50–60 °C. Following solidification, any excess agarose was carefully eliminated using a sharp surgical knife.

### Tissue Clearing Through our Modified iDISCO‐CUBIC protocol—RI Matching

The tissue blocks that have already been embedded are subjected to methanol dehydration. The samples were dehydrated at room temperature in 20%, 40%, 60%, and 80% methanol for 2 h and then incubated in 100% methanol for 2 h twice, followed by a solvent exchange to DCM/MeOH (2:1). Until they sank at the bottom of the vial, solvent exchange to 100% DCM for 1 h twice to wash out the methanol. Finally, samples were incubated (without shaking) in DBE and refreshed the solvent every 12 h until the tissues were no longer clear. The samples were stored in DBE at room temperature.

### Human Samples Collection

Human tissues were collected in the sterile surgery room at the Xiang'an Hospital of Xiamen University. Fresh tissues of breast tumor were immediately transferred to germ‐free 5 mL conical tubes with sterile DMEM medium supplemented with 10 µm Cy5ADA. The tissue tubes were placed in a clean, sterile cell incubator at 37 °C aerobically with 5% CO2 for   h. All samples were collected and analyzed after informed consent was obtained from the patients. The use of patient specimens for this work was approved by the Xiamen University Medical Institutional Review Board under the following protocol numbers XDYX202310K63. The patient information of all human samples was provided in **Table** **4**.

**Table 4:** Breast tumor (BT) samples from four patients.

Specimen _ID	Patient ID	Sample type	Tissue Type	Analysis	Age	Gender	BI‐RADS	Pathology	Tumor stage
BT_01_A	P_BT_01	Fresh tissue	Breast tumor (BT)	Tissue sections	43	Female	4C	invasive ductal carcinoma	pT2N2M0, IIIA, luminal B1
BT_02_A	P_BT_02	Fresh tissue	Breast tumor (BT)	Tissue clear for 3D imaging	65	Female	5	Invasive ductal carcinoma	cT4N1M1, IV, luminal B
BT_03_A	P_BT_03	Fresh tissue	Breast tumor (BT)	Tissue clear for 3D imaging	42	Female	4C	Invasive ductal carcinoma	T2N0M0, IIa, luminal B1
BT_04_A	P_BT_04	Fresh tissue	Breast tumor (BT)	Tissue clear for 3D imaging	58	Female	5	Invasive ductal carcinoma	pT2N1M0, IIB, luminal A

### Quantification and Statistical Analysis

Asterisks in the figures indicate the level of statistical significance (^*^ *p* < 0.05, ^**^ *p* < 0.01, ^***^ *p* < 0.001) as determined using a two‐tailed unpaired Student *t*‐test as defined in figure captions. Tests were performed using GraphPad Prism software (Version 8, Graphpad Software, La Jolla, CA, United States). Data are expressed as mean ± SD unless otherwise stated.

## Conflict of Interest

The authors declare no conflict of interest.

## Author Contributions

Y.W., Z.J., K.Z., and H.T. contributed equally to this work. Y.W., C.Y., and X.D. conceived the project. Z.J. performed tissue clearing and 3D imaging experiments, J.G. performed thin tissue section experiments. G.H. and B.L. performed cardiac perfusion for mice. K.Z. processed patient specimens for FDAA culture, K.Z., G.W., and H.T. provided patient specimens. Y.W., Z.J., and L.L. contributed to data analysis and interpretation. Y.W. and X.D. wrote the manuscript with comments from all authors. All authors read and approved the final manuscript.

## Supporting information



Supporting Information

Supplemental Video 1

Supplemental Video 2

Supplemental Video 3

Supplemental Video 4

Supplemental Video 5

Supplemental Video 6

Supplemental Video 7

Supplemental Video 8

Supplemental Video 9

Supplemental Video 10

Supplemental Video 11

Supplemental Video 12

Supplemental Video 13

Supplemental Video 14

## Data Availability

The data that support the findings of this study are available in the supplementary material of this article.
