# Supplementary material for: Whole‐Tumor Clearing and Imaging of Intratumor Microbiota in Three Dimensions with miCDaL Strategy
Source: Adv Sci (Weinh). 2024 Oct 8;11(44):2400694. doi: 10.1002/advs.202400694 (PMC11600245; doi:10.1002/advs.202400694)
Supplement: Supplementary file 1 — Supporting Information [file ADVS-11-2400694-s005.pdf]

ADVANCED SCIENCE
Open Access

Supporting Information

for *Adv. Sci.*, DOI 10.1002/advs.202400694

Whole-Tumor Clearing and Imaging of Intratumor Microbiota in Three Dimensions with miCDaL Strategy

Yuezhou Wang, Zile Jiang, Kai Zhang, Huimin Tang, Guimei Wang, Jinshan Gao, Guanghui He, Baoyue Liang, Li Li, Chaoyong Yang and Xianming Deng**

Supporting Information

Whole-Tumor Clearing and Imaging of Intratumor Microbiota in Three Dimensions with miCDaL Strategy

Yuezhou Wang[a]+, Zile Jiang[a]+, Kai Zhang[b]+, Huimin Tang[c]+, Guimei Wang[d], Jinshan Gao[a], Guanghui He[a], Baoyue Liang[a], Li Li[a], Chaoyong Yang[e]* and Xianming Deng[a]*

This file includes:

Figure S1 to S8

Figure S1

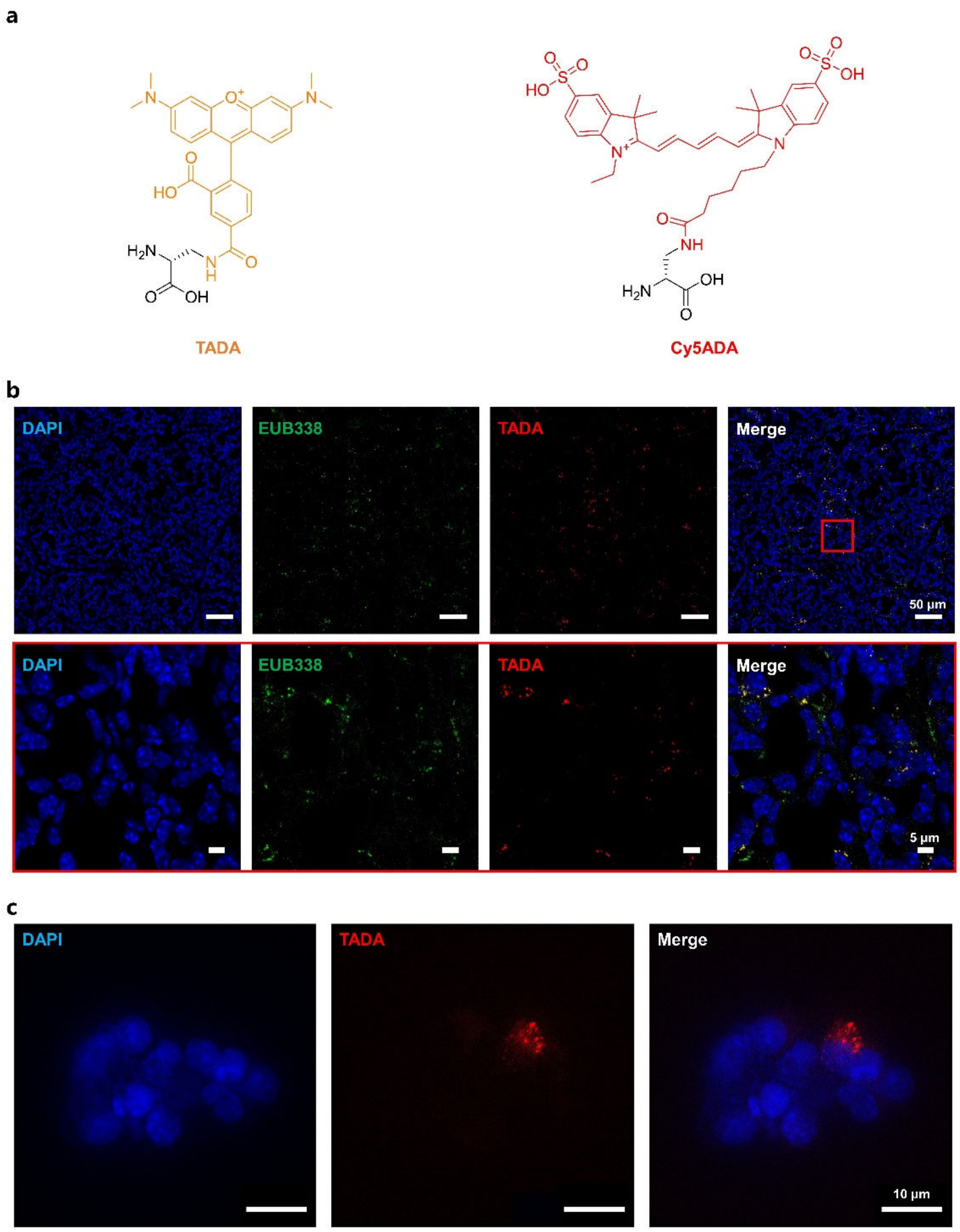

Figure S1. FDAAs label indigenous intratumor microbiota *in situ* through facile intratumoral injection

(a) Chemical structures of fluorescent D-amino acid derivatives. TADA (Orange), Cy5ADA (Red). **(b)** Tumor tissue section and **(c)** dissociated tumor cells analysis of indigenous intratumor microbiota from MMTV-PyMT mice. The mice were intratumorally injected with TADA, after 18 h of post injection, the tumors were harvested for analysis. EUB338 is a universal probe for bacteria 16S rDNA. Blue: DAPI, Red: TADA, Green: EUB338. For tissue sections, scale bars, 50 μm, magnified views, scale bars, 5 μm. For dissociated tumor cell, scale bars, 10 μm. Representative data from three independent experiments are shown.

Figure S2

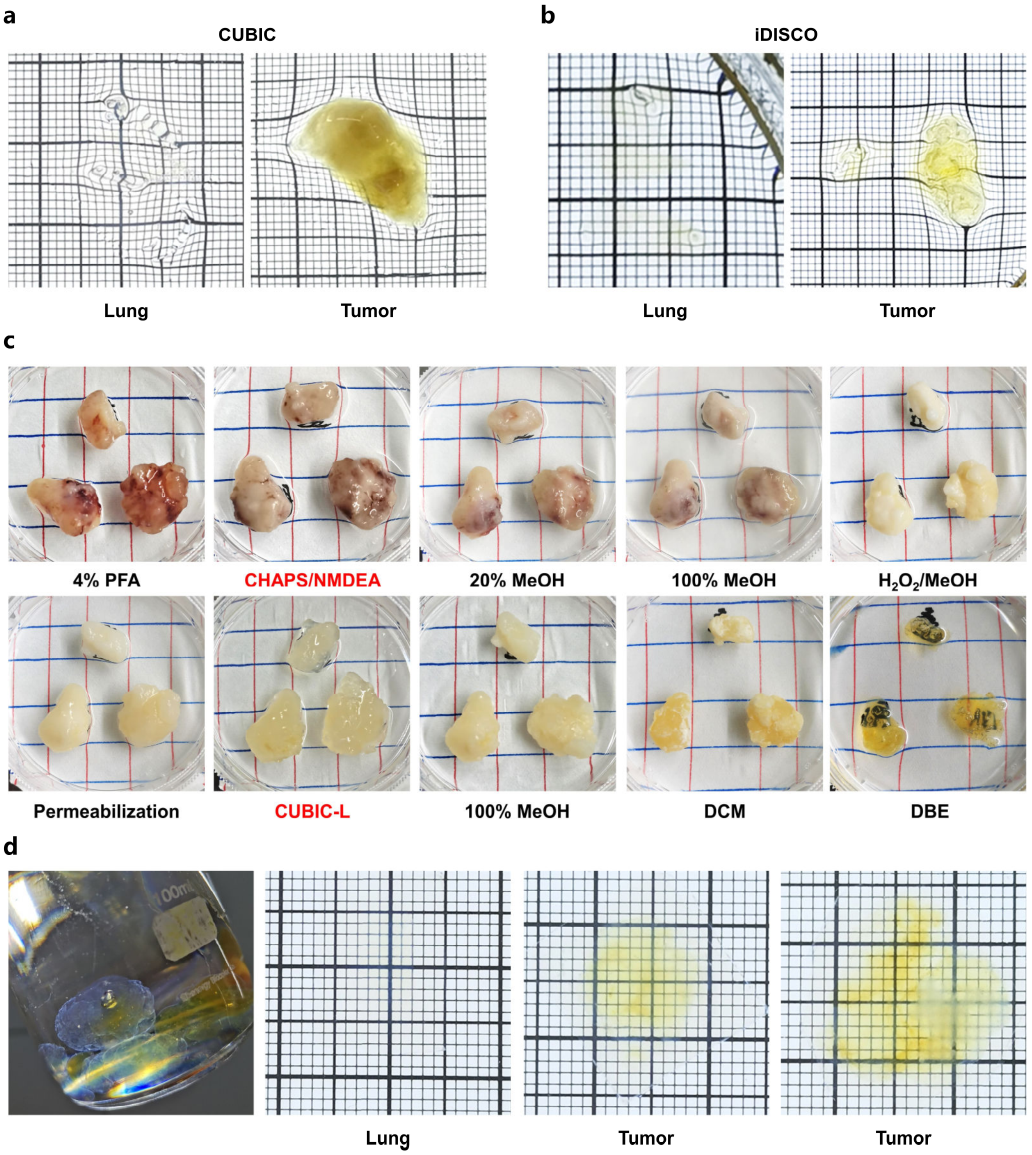

Figure S2. Comparison of the effects of different tissue clearing methods.

(a) Fixed lung and tumor tissues were treated with CUBIC. **(b)** Fixed lung and tumor tissues were treated with iDISCO. **(c)** Flow chart of our modified iDISCO-CUBIC tissue clearing protocol. CHAPS/NMDEA:3-[(3-cholamidopropyl)dimethylammonio]-1-propanesulfonate/N-methyldiethanolamine. CUBIC-L: Triton X-100/ N-Butyldiethanolamine, Permeabilization solution: Triton X-100/ deoxycholic acid/ heptakis(2,6-di-O-methyl)-β-cyclodextrin. **(d)** Fixed lung and tumor tissues were treated with iDISCO-CUBIC method. Lung and tumor tissues from MMTV-PyMT mice were sufficiently transparent using our modified iDISCO-CUBIC protocol.

Figure S3

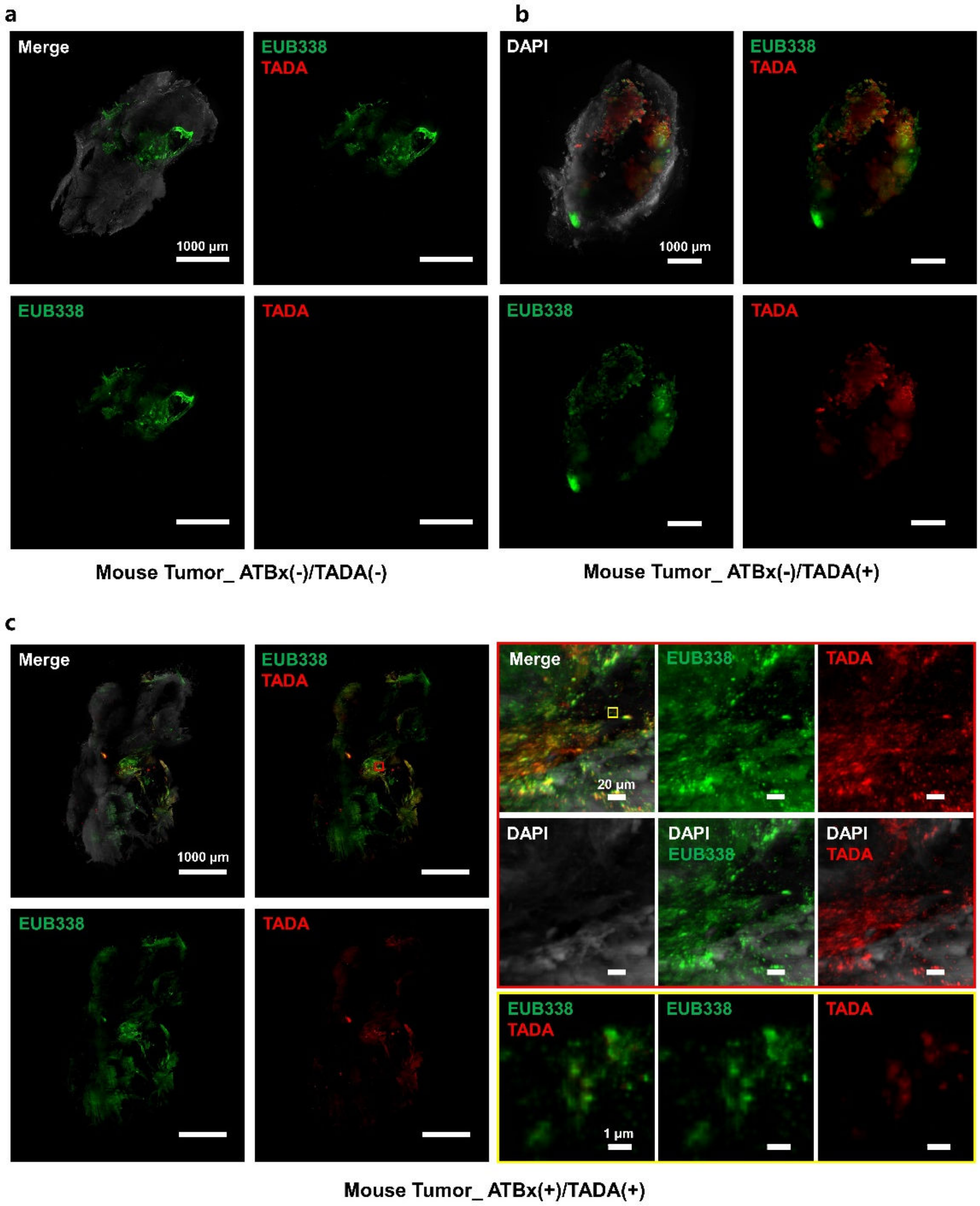

Figure S3. FDAAs specifically label intratumor microbiota in cleared tumor tissue in 3D views.

(a-c) 3D views of tumor tissues from PyMT mice that receiving various administration strategies of antibiotic cocktail (ATBx) and TADA. ATBx(-): no antibiotics treatment. ATBx(+): antibiotics treatment. TADA(-): no TADA injection, TADA(+) intratumoral injection of TADA. White: DAPI, Red: TADA, Green: EUB338. 3D views in **(a)-(c)**, Scale bars, 1000 μm. Magnified views in **(c)**, scale bars, 20 μm. Secondary magnified views in **(c)**, scale bars, 1 μm.

Figure S4

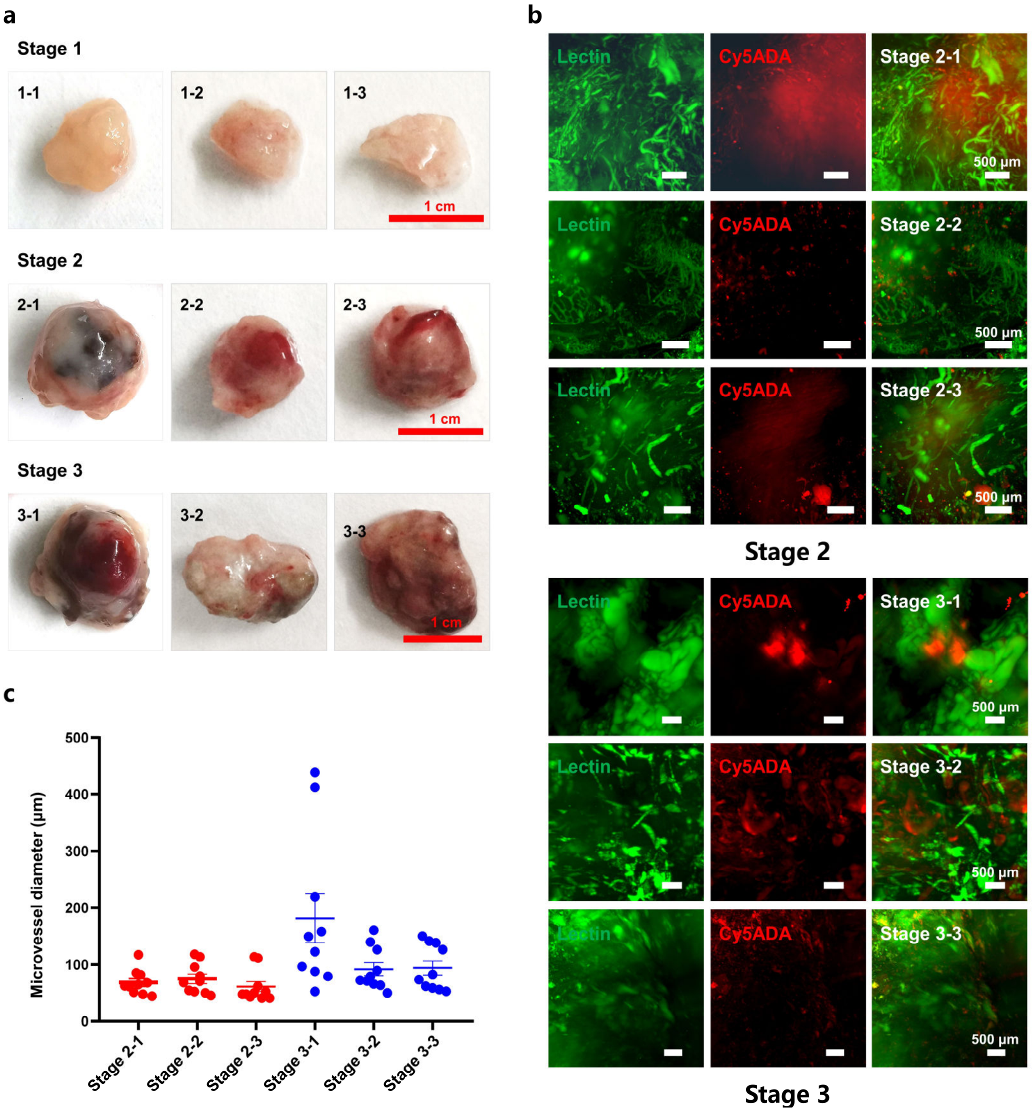

Figure S4. Intratumor microbes show preference for the necrotic region during tumor development.

(a) Pictures showing the appearance of tumors in different developmental stages from MMTV-PyMT mice. Tumors in stage 1 showed a white appearance, tumors in stage 2 and stage 3 displayed evident vascular invasion. **(b)** Magnified views of tumors in stage 2 and stage 3. The morphology of microvessels within these two tumors were clearly depicted. Green: Lectin, Red: Cy5ADA. Scale bars, 500 μm. N = 3, for each stage. **(c)** Comparison of the diameters of microvessels in tumors at stage 2 and stage 3 (10 microvessels from each tumor were measured). N = 3, for each stage.

Figure S5

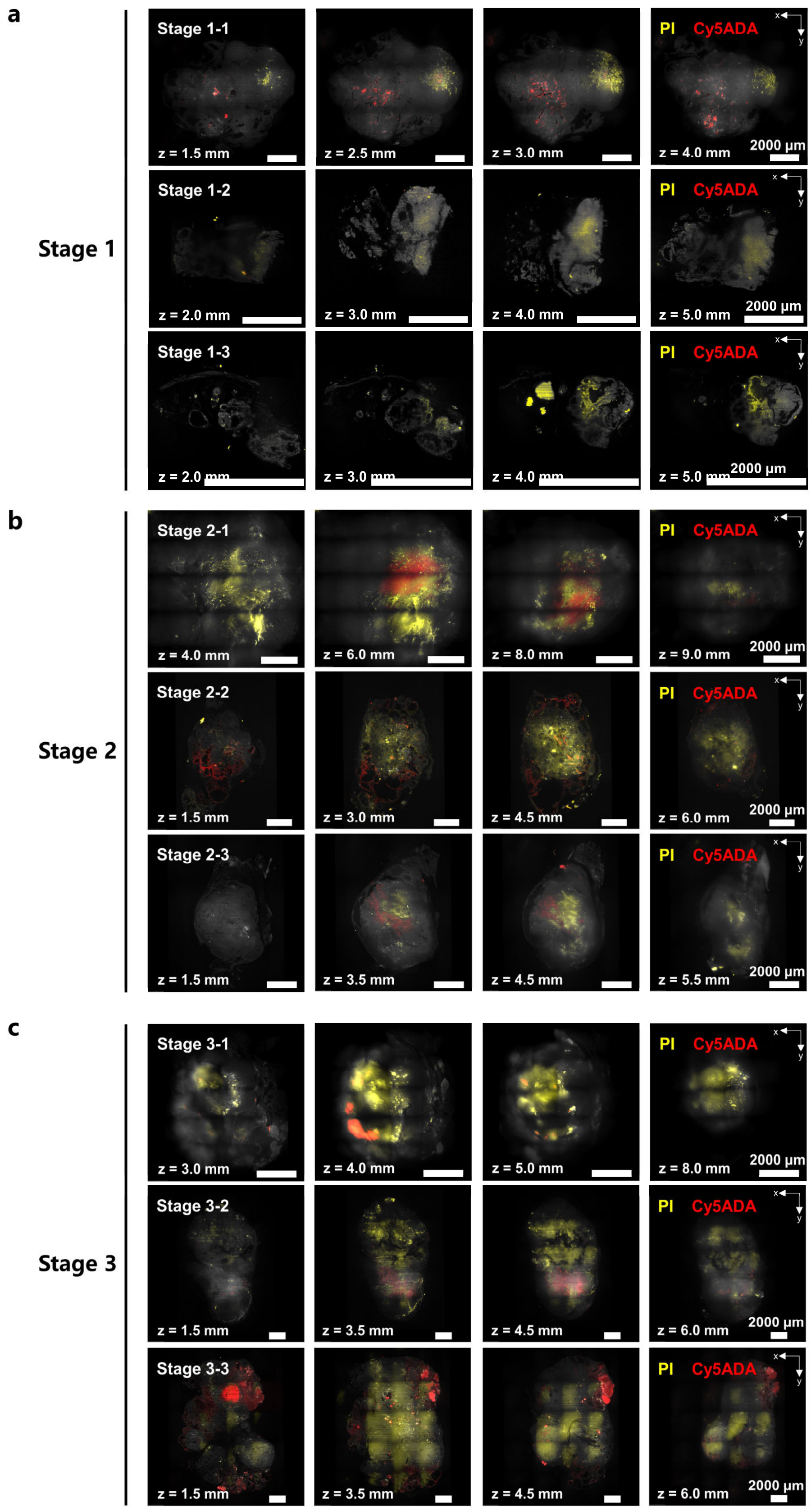
a
Stage 1
Stage 1-1
z = 1.5 mm
z = 2.5 mm
z = 3.0 mm
PI Cy5ADA
z = 4.0 mm
2000 μm
Stage 1-2
z = 2.0 mm
z = 3.0 mm
z = 4.0 mm
PI Cy5ADA
z = 5.0 mm
2000 μm
Stage 1-3
z = 2.0 mm
z = 3.0 mm
z = 4.0 mm
PI Cy5ADA
z = 5.0 mm
2000 μm
b
Stage 2
Stage 2-1
z = 4.0 mm
z = 6.0 mm
z = 8.0 mm
PI Cy5ADA
z = 9.0 mm
2000 μm
Stage 2-2
z = 1.5 mm
z = 3.0 mm
z = 4.5 mm
PI Cy5ADA
z = 6.0 mm
2000 μm
Stage 2-3
z = 1.5 mm
z = 3.5 mm
z = 4.5 mm
PI Cy5ADA
z = 5.5 mm
2000 μm
c
Stage 3
Stage 3-1
z = 3.0 mm
z = 4.0 mm
z = 5.0 mm
PI Cy5ADA
z = 8.0 mm
2000 μm
Stage 3-2
z = 1.5 mm
z = 3.5 mm
z = 4.5 mm
PI Cy5ADA
z = 6.0 mm
2000 μm
Stage 3-3
z = 1.5 mm
z = 3.5 mm
z = 4.5 mm
PI Cy5ADA
z = 6.0 mm
2000 μm

Figure S5. Intratumor microbes show preference for the necrotic region during tumor development.

(a-c) X-Y optical sections of PyMT tumors at stages 1-3, obtained at different Z-axis depths. White: Lectin, Yellow: PI, Red: Cy5ADA. Scale bars, 2000 μm. N = 3, for each stage.

Figure S6

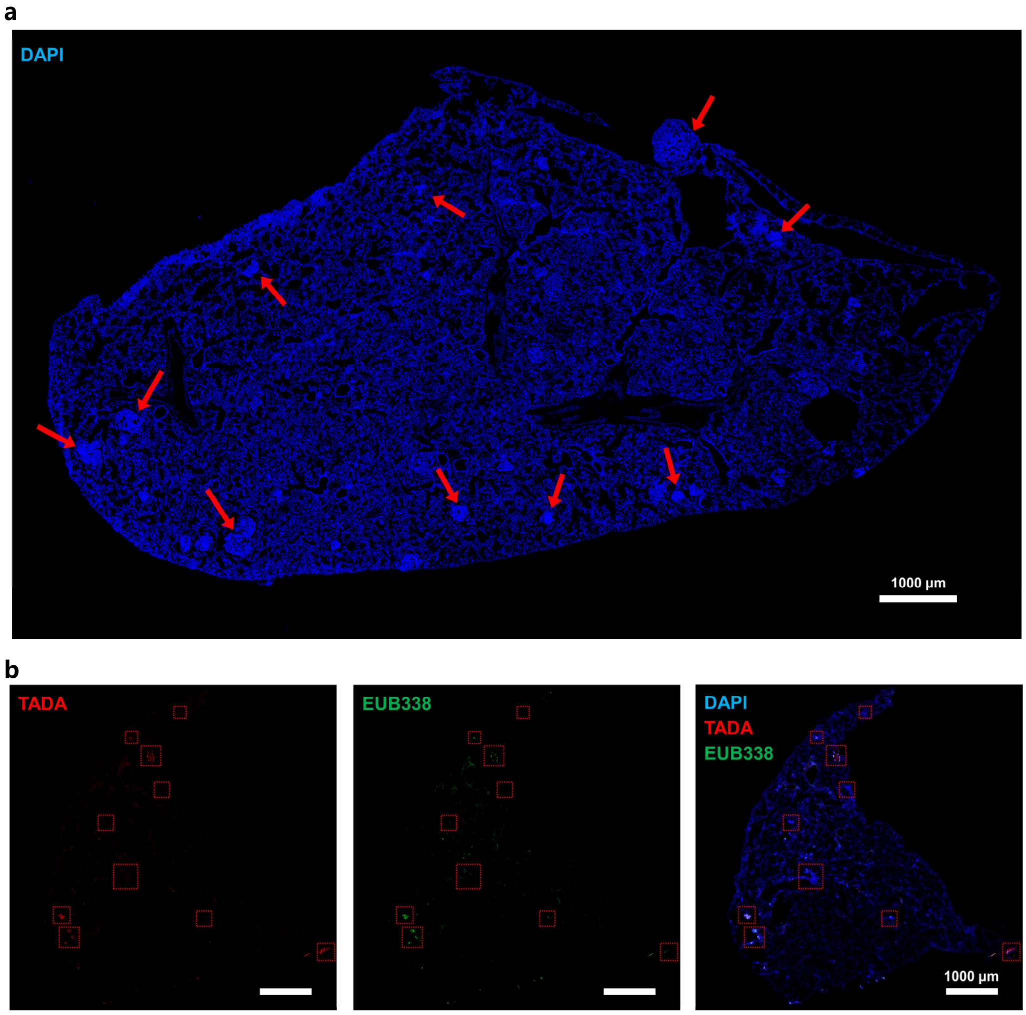

Figure S6. FDAAs label indigenous microbiome in lung through intravenous injection.

(a) Whole-mount lung tissue section imaging of MMTV-PyMT mice. Red arrows indicate metastatic foci, Blue: DAPI, Scale bars, 1000 µm.

(b) Whole-mount lung tissue section imaging of indigenous microbiota from MMTV-PyMT mice. The mice were intravenously injected with TADA, at 6 h of post injection, the lungs were harvested for frozen section. EUB338 is a universal probe for bacteria 16S rDNA. Red boxes indicate multiple metastatic foci. Blue: DAPI, Red: TADA, Green: EUB338. Scale bars, 1000 µm. In (a) and (b), representative results from three independent experiments are shown.

Figure S7

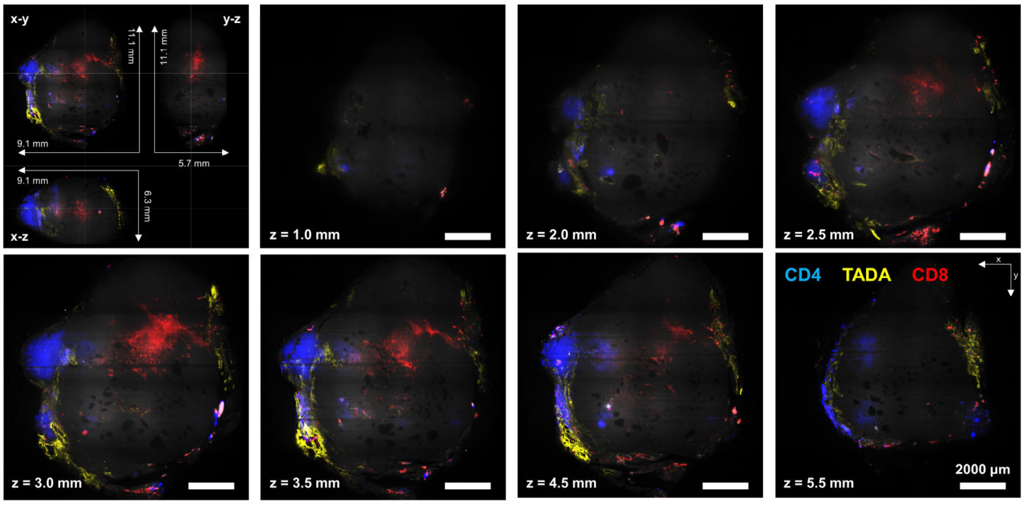

Figure S7. X-Y optical sections results of intratumor microbiota and activated T cells in tumors of MMTV-PyMT mice.

The view of the tumor from X-Y, X-Z, Y-Z direction, and X-Y optical sections obtained at 1.0, 2.0, 2.5, 3.0, 3.5, 4.5, and 5.5 mm in Z-depth. Activated T cells are indicated by CD4 and CD8 antibodies. Intratumorally injected TADA was used for *in vivo* labeling of intratumor microbes. Blue: CD4, Yellow: TADA, Red: CD8, White: DAPI. Scale bar, 2000 μm. Representative results from three independent experiments are shown.

Figure S8

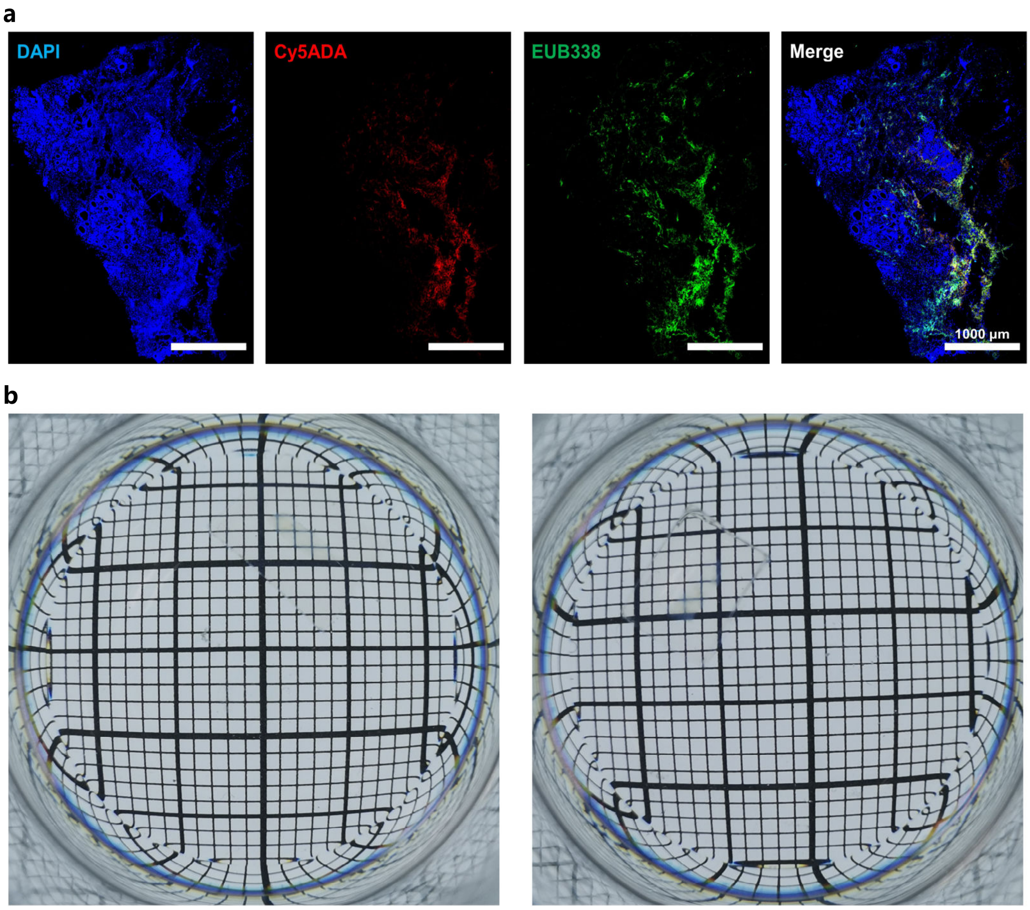

Figure S8. FDAAs label indigenous intratumor microbiome in human breast tumor tissues by simple incubation.

(a) Whole-mount human tumor tissue section imaging of indigenous microbiota. Fresh human breast tumor tissues were immersed in a sterile environment with Cy5ADA solution (10 μM, 37 °C, 5% CO_2, 1 h). The tissue sections were subsequently prepared and subjected to FISH hybridization using the EUB338 probe for precisely localizing bacterial distribution within the tissue slices. Blue: DAPI, Red: Cy5ADA, Green: EUB338. Scale bars, 1000 μm. Data are representative of at least three independent experiments.

(b) Human breast tumor tissues are sufficiently transparent using modified iDISCO-CUBIC method.
